# Suicide and filicide in postpartum psychosis

**DOI:** 10.1007/s00737-016-0675-8

**Published:** 2016-10-24

**Authors:** Ian Brockington

**Affiliations:** University of Birmingham, Edgbaston B15 2TT, Birmingham, UK

**Keywords:** Suicide, Filicide, Childbearing psychoses, Organic psychoses, Postpartum psychoses

## Abstract

This paper reviews the frequency of suicide and filicide in a literature of over 4000, and personal series of 321, childbearing psychoses. Suicide is rare during the acute episode, but the rate is high later in the mother’s life and in first degree relatives. The filicide rate is high in depressive psychoses (4.5 %), but lower in episodes without overt depression (less than 1 %), and some of these appear to be accidental, without intent to kill.

## Introduction

Both suicide and filicide are uncommon events in puerperal psychosis. This article deals with psychosis, not postpartum depression; but according to the best epidemiological studies, the overall suicide rate is relatively uncommon in the year following childbirth, in spite of the long list of severe psychological disorders that develop in the puerperium (Appleby et al. 1998; Gissler et al. 1996). The risk of filicide has also been exaggerated (Brockington 2016).

## Suicide

### Completed suicide in follow-up studies

Fifteen studies have provided data on suicide. Most had a mixed sample, including depression without psychotic features. Collectively they reported 46 suicides in about 15,000 person/years, that is about 300/10^5^ person/years. This figure is much higher than the highest national rates for women (La Vecchia et al. 1994); Hungary, near the top of the list, had 17/10^5^. It is much influenced by one study (Schöpf 1992), which found 13 cases among 119 mothers. A Dutch thesis (Klompenhouwer 1992) is unique in calculating a precise suicide rate (113/10^5^ person/years), which was 17 times the rate for women in the Netherlands (6.5/10^5^ person/years). These figures, however, report lifetime risks, not suicidal outcomes of acute psychotic episodes. Schöpf reported a mean survival of 13 years after the index episode, with the earliest suicide 2 years afterwards.

### Suicide in childbearing psychoses

Excluding parturient suicide, whose cause is not psychosis, but the ordeal of labour, there are, among over 4000 cases of childbearing psychosis (described in detail), only two—cases of completed suicide during the index episode. One, mentioned only briefly, was a post-abortion psychosis occurring 2 months after an extra-uterine pregnancy (Ferguson 1892). The other probably occurred during an infective delirium (Pollák 1929):

A doctor’s wife, herself a doctor, gave birth to a stillborn child. She had rigors and developed a fever of 39.5°, which reached a peak of 40.6°. She became restless, and expressed persecutory ideas. On day 11, in an unguarded moment, she jumped from the window to her death.

In my legal work, I encountered one postpartum suicide associated with depressive psychosis:

A 25-year old soldier’s wife was delivered of her 1^st^ child by emergency Caesarean section, because of foetal distress. The baby required phototherapy for jaundice, and the mother developed a fever, which soon settled with metronidazole. A week later she became depressed, and on day 16 was hearing voices instructing her to commit suicide. She drank toilet disinfectant, filled a bath to drown herself and tried to strangle herself with the flex from her iron. She told a psychiatrist, “I’ve got to do it”. In letters discovered after her death, it was clear that she thought she had cancer. “You don’t know how happy it would have made me (if I killed myself), because my brain was full of it. People like me they put in a padded cell, where you just lay there until you die. I pray you are reading this, and know that I am at peace”. There were words of love, and a remark about how lovely the baby was. To her mother, she wrote: “I don’t want to die and love you all, but I am going mad”. On day 22 she disappeared. Three days later she was found hanging from a tree.

In the literature, five suicides occurred later in the mother’s life, after recovery from the postpartum episode (Winter 1908; Frumkes 1934; Beckmann 1939; Callieri 1955; Trixler et al 1981). A mother in my series, with bipolar disorder, who suffered two early postpartum depressive psychoses, threw herself from a bridge some years later.

### Suicide in first degree relatives

There is a strong family history of suicide in these mothers. In the literature, 18 lost fathers, eight lost mothers (one after her own birth), six lost brothers and three lost sisters, one of whom lost a twin sister who had just given birth to twins; one lost both father and brother, and one lost father, mother and a brother. In my series of 321 childbearing psychoses, 13 lost 1° relatives—four a father, three a brother, four a sister, one a ‘sibling’ and one her mother after her own birth. With one exception, all of these mothers had a lifetime diagnosis of bipolar or cycloid; this exception had six episodes of a recurrent brief psychosis, of which two episodes had premenstrual onsets (possibly a bipolar/cycloid variant).

## Filicide

### Overall frequency

An unpublished compilation of 800 published cases of filicide included 65 due to depression or psychosis within a year of the birth. Eleven were depressed without psychotic features. One had a chronic psychosis and 12 had insufficient evidence either of depression or psychosis, leaving 48 with evidence of psychosis^1^—just over 1 % of the 4029 cases reviewed. Recent American papers have given the much higher figure of 4 % (Spinelli 2009; Hatters-Friedman and Sorrentino 2012). This error can be traced to a misreading of a Scottish study (Davidson and Robertson 1985), in which 82 patients were followed for a mean of 16 years: only 29 were suffering from bipolar, paranoid or schizophrenic psychoses, the rest from depression; one definite and one probable filicide were committed by depressed mothers, and a mother probably suffering from a chronic psychosis killed two children. None of the filicides was committed by a mother suffering from an acute psychosis without depression.

The literature contains this tragic South African case, remarkable for the killing of five infants (Allwood 1992):

A 25-year old gave birth to twins, who were admitted for phototherapy to treat neonatal jaundice. The mother was a lodger, and was noticed collecting shoes and placing them in lines. One evening she went into the neonatal intensive care ward, grabbed her twins and threw them against the wall, then killed three other babies. She has since had further children without mental illness.

### Filicide in organic psychoses

Six cases occurred during organic psychoses; since 1,368 organic psychoses have been reported in the literature (Brockington 2014), this is 1/228. This is an example of filicide during an infective psychosis (v Krafft-Ebing 1875):

A 26-year old single woman concealed her pregnancy and gave birth alone. She developed puerperal fever, and on day 13 became disturbed, singing and shouting. In this state she strangled her child, said so, and immediately began singing and roving around again. Peritonitis set in, but she recovered with no memory for the deed.

Two filicides occurred during epileptic psychoses (Hoppe 1893; Quensel 1907):

A patient suffered from seizures in childhood and then menstrual epilepsy. After giving birth to her first child she had fits and *Tobsucht;* on the first night she threw her child into the yard, where it was eaten by dogs.

An epileptic woman had several similar attacks of *Dämmerzuständen* [twilight states] immediately after delivery; in the last of these she threw her two children out of a 4^th^ storey window.

### Filicide in depressive psychoses

Most of the non-organic filicides occurred during depressive psychoses—three with prepartum onset, ten with early postpartum onset and 11 with late postpartum onset. This is a famous case of early onset melancholia, leading to filicide on day 13 (Leader 1848):

A 37-year old showed signs of derangement – she was going to die and would go to hell. She was ‘prostrated of strength’, her eyes vacant and wild, her countenance haggard. The doctor gave instructions that she should not be left alone, and should not be given the child. But, 13 days after the birth, she angrily demanded that her teenage daughter bring it to her. She nearly decapitated the baby with a razor.

In the following cases, filicide was an issue after two births (Matheson 1941; Fallgatter et al 2002):

A 24-year old gave birth to her 1^st^ child. Within the first three weeks she became depressed and had impulses to kill the child although she loved it very much [obsessions of infanticide]. Two years later she gave birth to her 2^nd^ child. On day 20 she became depressed, said her brain was gone, she could not concentrate or remember anything and thought she was going to hurt the child. During a failure of supervision, she cut its throat, then her own. On admission to hospital she was confused; she could not remember killing the baby, only seeing herself in the mirror with her throat cut.

A 29-year old woman from Kazakhstan, whose father hanged himself, gave birth to a child, and within a month became depressed. She tried to suffocate the infant, but was prevented by the family. Ten years later they came to Germany. She gave birth again. On day 5 she became ill, with a staring expression. On day 10 the baby was found drowned in the bath. Admitted to hospital she was in a perplexed and sub-stuporose state with ideas of guilt and punishment, and auditory hallucinations; she heard the baby cry; it had a bad mother and a bad future.

In the next case, four children were killed (Dolenc 1913):

A 32-year old, with a sick husband, became depressed and asked him to kill her - she was a great sinner. She cut the throats of all four children, tucked them up in bed, lit candles so that they would not be in darkness, and disappeared. She was found wandering three days later. In prison, she lay on the floor, sleepless, without the strength to kill herself. She had no sympathy for others’ grief, having no love left in her heart.

Melancholic filicides, often accompanied by suicidal actions, are a notorious risk in severely depressed mothers. Twenty-four cases among 535 depressive psychoses is 1/22 (4.5 %).

### Filicide in postpartum psychoses without depressive features

The remaining cases occurred during postpartum psychoses without overt depression. There were three with late postpartum onset and five with unknown postpartum onset. In one, there were no details of the psychopathology, which may have been depressive; suppression of an unwanted child is an alternative diagnosis (Hume 1797):

In 1756, Agnes Crockat, an unmarried woman, called for help with her delivery, but a week later became strange in speech and behaviour, and strangled the child. She kept it beside her on the bed, and, when visitors came, said ‘the Devil had tempted her’. She was sentenced to death, but pardoned by Royal Mercy.

Nine cases with early onset psychoses will now briefly be summarized.

In the next five, there may have been no *mens rea*—no intention to kill, the baby dying accidentally (Trigueros 1906; Chevalier-Lavoure and Voivenel 1910; Gosselin and Bury 1969; Dayan 1977; Moisan 1982):

A 25-year old, whose mother suffered from depression, gave birth to her 1^st^ child. From that time she became excited. She left home at 2 am, naked, and made her way to a river, where the infant was carried away. After her arrest she was sleepless and anorexic and completely indifferent to people and her own injuries.

A 23-year old, with a history of two psychotic episodes, gave birth to her 1^st^ child: afterwards she became sleepless, talking and gesticulating. On day 4 she became violently agitated and suffocated her infant by forcing a thimble into its mouth. Admitted to hospital, she did not know where she was and appeared not to recognize anyone. She was terrified by visual and auditory hallucinations, fought and tried to escape, colliding with doors, walls and furniture that she appeared not to see. She lapsed into stupor, mute, cataleptic and resistant to all approaches. From time to time she took on an ecstatic attitude, as if in prayer. Mysticism gave place to eroticism. Three months later she recovered, with amnesia for her illness, which seemed like a dream.

A 20-year old, whose father suffered two ‘affective episodes’, gave birth to her 1^st^ child. On day 9 she developed a confused and dream-like episode. Six hours later she covered her daughter with talcum powder, inhalation of which caused her death. She spoke of the end of the world and said she covered the child with powder to prevent its freezing or to reanimate it after death. She danced with the child, not realising it was dead. She told her husband they could have other children, or adopt them. She was in hospital for 15 days but showed little sign of illness.

Two days before giving birth, a woman began to have lacunae in her memory, which persisted until the explosion of a postpartum psychosis with illusions and hallucinations. She drowned her infant while bathing it, then seized the dead baby and showed it to a neighbour in a state of morbid exaltation.

A woman gave birth to her 1^st^ child: 17 days later, she became agitated and sleepless. For four days she walked about her apartment, breast-feeding the infant, although she had no milk. On the 4^th^ day the child died, apparently of neglect. Admitted to hospital, she was in a state of manic agitation, confused with auditory and visual hallucinations and illusions. She believed her husband had died, God had told her the baby would be resuscitated, she was still pregnant and would give birth at the end of the year.

These are the remaining four, in whom filicide seemed deliberate (Philippi 1830., Ketai and Brandwin 1979; Colasson et al. 1981; Spinelli 2009):

14 days after childbirth, a woman began to have ideas of sin, then became more disturbed with swearing, violence, seeing the Devil, dancing with raised skirts, saying obscene things, and singing in a crazy tone. In the night she stabbed her three children, and was about to stab her husband when he woke and overpowered her.

At the age of 17, a woman developed auditory hallucinations and persecutory delusions. At 24 she gave birth to her 1^st^ child. Three weeks later she became agitated, saying that her child was controlled by the Devil. She strangled it and took an overdose.

A woman became depressed after giving birth to her 1^st^ child. After her 2^nd^ birth, on day 4, she developed a *bouffée délirante* with exaltation, hallucinations and themes of guilt and religiosity. She killed the child (no details).

A 34-year old suffered from a periodic disorder with three days of jocularity, creativity and high energy alternating with tearfulness and withdrawal. She gave birth to her 2^nd^ child: on day 2 she became sleepless and suspicious that her husband would harm the baby. She had thoughts of throwing him out of the window, believing he had gas inside him. She became agitated and confused, and could not think straight. On day 20 she felt as if she was being taken over by ‘a force’: she did not feel connected to her hands. In a dazed, trance-like state, she strangled her baby with a telephone cord, then attempted to cut her wrists.

The frequency in early onset psychoses, including accidental filicides, is 9/1,115 (less than 1 %). The risk would be even lower, once the mother had come under medical care.

### Filicide in my series

Among 321 childbearing psychoses, there were two completed filicides, both occurring during depressive psychoses. The first mother suffered from bipolar disorder and had several episodes related to subsequent pregnancies; at the time of the filicide, she was depressed, without psychotic features:

A 23-year old gave birth to her 1^st^ child. She was discharged from the maternity hospital after ten days. She was attached to the baby, but felt ‘really low’ and would cry, cry and cry and bang her head against the wall. The baby took two hours to feed and required another feed 1½ hours later. She was “so tired” and had no family to help. She ate nothing and her weight went down from 10 to 7½ stones. “I felt like pulling out my hair, I was that bad”. After seven days at home she ‘just smothered the baby’. “I must have thought, ‘for Christ’s sake shut up’ and put a pillow over him”. Her husband found the baby dead, and his wife gone. She did not remember the act, and could not believe she had done it.

The second mother killed her 4½-month old baby in the context of a postpartum depressive psychosis.

After a pregnancy complicated by pre-eclamptic toxaemia, a 31-year old was delivered of her 2^nd^ child by emergency Caesarean section @ 34 weeks gestation. She became depressed, losing one stone in weight. Two months later she started to worry about her baby’s health. She asked for blood tests and a brain scan. She believed he was very cold and in danger of hypothermia. She became obsessed with these ideas and was continually checking on him. A letter from a physiotherapist about a minor hip problem was interpreted as removing him because of maltreatment, causing brain damage. She could not be reassured, and “unbearable pressure” built up in her head. When the baby was 4½ months old, she drowned him in the sink, and cut her neck and wrists. She was ill for a year but recovered after ECT and successfully mothered another child.

I had no filicides associated with puerperal psychosis without depressive features, but there were dangerous assaults. The following mother endangered her infant’s life without filicidal intent:

A 21-year old developed puerperal mania. She made a rapid recovery but relapsed at her 1^st^ menses. She was arrested by the police, trying to stuff an apple into the baby’s mouth. It had begun to turn blue, its throat blocked by apple and banana; a policeman saved its life by mouth-to-mouth respiration.

This mother took a knife to her 18-month old son in a recurrence of a cycloid episode:

A 28-year old gave birth to her 1^st^ and only child; this was followed by a cycloid episode, starting on day 1. Eighteen months later she became depressed, withdrawn and preoccupied, ‘on a downward spiral’. One night she was restless; at dawn she saw vapour trails in the sky, which she thought were angels. She said, “It’s the end of the world. Look at the clouds”. She appeared confused, preoccupied and distressed, holding her head and screaming. She was afraid devils would take her son away. At 5.30 am she attempted to sacrifice him, stabbing him in the chest five times with a large kitchen knife, penetrating the skin, but causing little damage. She was shouting, “I’ve got to kill the Devil’s baby and free the world”. Her husband disarmed her, suffering wounds to his hands. She telephoned a friend and said, “I’ve done it. I’ve killed the Devil’s baby, like you told me to”. As the baby was being taken to hospital, she grabbed the ambulance doors and said, “Don’t let them take him. They will kill him”; she had to be wrenched away from the door. While waiting for a doctor, she asked her husband to kill her. During the next 20 years she suffered several more episodes, all of which were thought to be premenstrual.

Another mother stabbed her 13-year old, and attempted to immolate herself and her 6-week old baby, when in the grip of a paranoid psychosis:

A 37 year old, in an unstable relationship, was delivered of her 4^th^ child by Caesarean section @ 36 weeks gestation. After the birth she was sleepless and anorexic. She took St John’s Wort for depressive symptoms, and smoked an unknown amount of cannabis. At five weeks postpartum she confided in an aunt that she believed the child’s father was trying to abduct the baby and take it to Spain. She expressed the idea that he had been tape-recording her, to gather evidence about her mothering. A week later, after two sleepless nights, she made incoherent, rambling telephone calls to her aunt and the police, implying that others were involved (organized crime, ‘a sick and perverted syndicate’), naming individuals: the children would be introduced to an internet paedophile ring and, before death, would suffer horrific sexual abuse and torture. She had a knife ready to protect her family, and a bucket of bleach to throw at an intruder. When the police arrived, she waved them away, believing they were imposters. After gazing at her sleeping 13-year old for half-an-hour, she stabbed her in the chest with a 5-inch knife. Her intention was to kill her, but when she woke and cried out, she came to her senses. She then cut her own wrists and neck. At 5.30 am the terrified children were running into the garden in the freezing cold. There was smoke coming from a window in a top room, where she had set light to the duvet to kill herself and the baby. Her daughter had two quarter-inch wounds on her left nipple and sternum, a punctured lung and pneumothorax. At the police station, the mother said, “I wanted to kill them, so that they would not suffer the pain I have suffered”.

The following mother attacked her 4-month old child in the context of a postpartum depressive psychosis with a menstrual relapse, which had manic features:

A 24-year old gave birth to her 2^nd^ child @ 38 weeks gestation. Within two weeks she became depressed, and lost two stones in weight. She developed ideas that people were talking about her, and the family was plotting against her. Four months after the birth she perceived that her son’s eyes were green, like the Devil’s eyes, and his voice had changed. She believed the Devil was trying to take him. She shook him, “tried to poke the Devil out of his eyes” and banged his head against the refrigerator, shouting for the Devil to leave him. “When I attacked my son, I was actually protecting him. I thought I was one of God’s messengers, and was getting a message from God, saying that everybody was evil except the angels”. The baby suffered severe injuries, including swollen eyes and mouth, lacerations to his gums and an eye, leaving scarring. Admitted to hospital the mother was high, irritable and suspicious, and showed pressure of speech and flight of ideas. She attacked the doctor and nursing staff, thought she could fly, and attempted to baptise other patients. She washed repetitively and ritualistically, gargling to the point of choking. The next day she lay in bed and refused to speak, then hit her head on a brick wall and had to be prevented from jumping out from a 3^rd^ floor window. Her menses started and, within two days, she improved, apologised, full of remorse, and was soon discharged well.

A mother, who suffered from a delusional depression of late postpartum onset, clearly intended combined suicide and filicide, but was frustrated by the fortuitous intervention of a stranger:

A 34-year old gave birth to her 2^nd^ child, and, five weeks later, developed a depressive psychosis, with delusional ideas about the infant. After four months she deliberately dropped her baby on the floor. Admitted to the Mother & Baby unit, she dissimulated her suicidal intentions. Six months after the birth, she drove for an hour, bought a hose-pipe, looked for a deserted place, drove into a field, attached the hose pipe to the exhaust, and locked the windows, leaving the engine on. A farm worker saw her, and rescued them.

There was one mother, who attacked her baby when in the grip of a psychosis of early postpartum onset:

A 26-year old was delivered of her 1^st^ child, by ventouse under epidural anaesthesia; this was followed by a renal infection. During labour she experienced auditory hallucinations. During the next two weeks she developed a system of persecutory ideas involving her sister, who had bugged her home, and wanted to kill her and the baby. She taped the letter-box to prevent her sister passing a letter bomb. She asked for more locks and closed circuit television, in case her sister broke in at night. She misidentified pedestrians as her sister in disguise. She became tirelessly overactive, starting a new business, organizing holidays and spending £1,000 on mail-order goods. She believed she was destined to become an amazing artist. After four weeks she came to believe her sister was living in the garage, or in a cupboard under the stairs. Early one morning she telephoned her father to warn him that her sister wanted to kill their mother too, using a public phone booth because she believed the police had bugged her telephones as well as the baby monitor, and were watching the house. A voice told her to kill the dog and cat, her sister and herself, so that they would all go to Heaven. That evening she telephoned for an ambulance, saying, “It is suicide … My sister tried to kill me. I am afraid I have had to kill her in self defense, because I have got a young baby here.” When the paramedics arrived, they heard screams and inarticulate rantings. She was kneeling on the floor over a child, compressing its chest with an 8-inch knife, rocking forward and pressing down. When a paramedic seized the knife, she said, “Kill me quickly”. The baby sustained two one-inch wounds in the chest, with no damage to underlying organs. The patient was in hospital for several months, made a complete recovery, had another child and remained well for 13 years.

Thus, in my series, all the attempted and completed filicides occurred in the context of depressive psychoses, but major assaults (one accidental) occurred during early onset postpartum psychosis, and in subsequent episodes.

## Conclusion

The suicide rate is high in the families of mothers with postpartum psychosis, and in subsequent psychotic episodes, but not during acute puerperal manic/cycloid episodes. Filicides are also uncommon during acute postpartum psychoses without depressive features.

## Footnotes

1 All 48 cases have been tabulated in *What is Worth Knowing about Puerperal Psychosis* (Brockington 2014).
